# The TDDFT Excitation Energies of the BODIPYs; The DFT and TDDFT Challenge Continues

**DOI:** 10.3390/molecules26061780

**Published:** 2021-03-22

**Authors:** Adrien Schlachter, Alexandre Fleury, Kevin Tanner, Armand Soldera, Benoit Habermeyer, Roger Guilard, Pierre D. Harvey

**Affiliations:** 1 Département de Chimie, Université de Sherbrooke, 25000, Boul. de l’Université, Sherbrooke, QC J1K 2R1, Canada; adrien.schlachter@usherbrooke.ca (A.S.); Alexandre.Fleury@USherbrooke.ca (A.F.); kevin.tanner@usherbrooke.ca (K.T.); Armand.Soldera@USherbrooke.ca (A.S.); 2 PorphyChem Porphyrin Chemicals & Engineering, 21000 Dijon, France; b.habermeyer@porphychem.com (B.H.); Roger.Guilard@u-bourgogne.fr (R.G.); 3 Institut de Chimie Moléculaire de l’Université de Bourgogne, ICMUB, Université de Bourgogne Franche-Comté UMR CNRS 6302, F-21078 Dijon, France

**Keywords:** BODIPY, DFT, TDFT

## Abstract

The derivatives of 4,4-difluoro-4-bora-3a,4a-diaza-s-indacene (BODIPY) are pivotal ingredients for a large number of functional, stimuli-responsive materials and therapeutic molecules based on their photophysical properties, and there is a urgent need to understand and predict their optical traits prior to investing a large amount of resources in preparing them. Density functional theory (DFT) and time-dependent DFT (TDDFT) computations were performed to calculate the excitation energies of the lowest-energy singlet excited state of a large series of common BODIPY derivatives employing various functional aiming at the best possible combination providing the least deviations from the experimental values. Using the common “fudge” correction, a series of combinations was investigated, and a methodology is proposed offering equal or better performances than what is reported in the literature.

## 1. Introduction

The BODIPY pigment and its derivatives are key entities for phototheranostics [1], including photodynamic therapy [2], functional optoelectronic materials [3], such as solar cells [4,5,6] and light emitting diodes [7], and stimuli-responsive materials [8,9,10,11]. In order to understand or predict the optical properties [12,13] of such important chromophore, and notably the lowest energy electronic transition, a very large number of investigations involving computational argumentations were reported but most of the time the correspondence between the calculated position and experimentally observed one turned out to be chronically poor, where differences ranging from 60 to 100 nm were commonly depicted [14,15,16,17,18,19,20,21,22,23,24,25,26,27]. However, on some rare occasions, the comparison between computations and experiments appeared much better [28,29]. In front of this curious phenomenon, multiple theoretical investigations were undertaken [30,31,32,33,34,35,36], and emphasis on the computational method, types of the basis sets, the importance of the solvent field, the use of excited state molecular dynamics, and even empirical corrections, were made [37,38,39,40,41,42,43,44,45,46]. Ab initio calculations were also reported with good results, but the computational time is also an unneglectable parameter to consider [47,48].

From all these previous investigations, the main conclusion is that the methodology that requires the least resources in material science and biomedical research is the application of an empirical correction: “*fudge*” [18,38,49]. Fudge methods generally consist in applying an empirical correction to calculated data to make them fit with experimental results. Usually, this method is used for TD-DFT computations where the shape of the simulated spectra compare favorably with the experiments but exhibit large offsets in terms of wavelength position.

**Figure 1:**
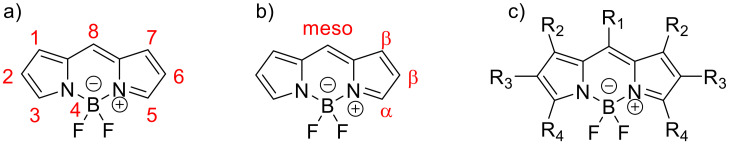
4,4-difluoro-4-bora-3a,4a-diaza-s-indacene (BODIPY) core. (**a**), IUPAC (International Union of Pure and Applied Chemistry) numbering. (**b**), alpha, beta, and meso designation. (**c**), investigated structures.

This work proposes a revisit where new basis sets and computational methods are applied to find the best combination in order to provide a better agreement between computations and experiments. Figure 1 depicts the structure of the BODIPY core and its classical numbering scheme, for the purpose of this paper, the alpha, beta, and meso designation will be used.

## 2. Investigated Structures

**Figure A1:**
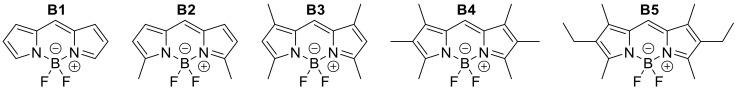
Hydrogen atoms placed at the meso position.

**Figure A2:**
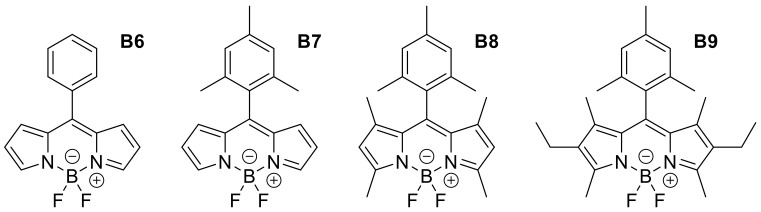
Aromatic groups placed at the meso position.

**Figure A3:**
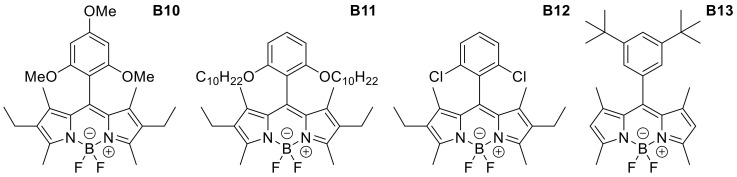
Substituted aromatic groups placed at the meso position.

**Figure A4:**
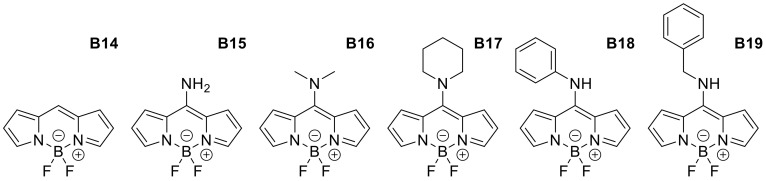
Amino groups placed at the meso position.

**Figure A5:**
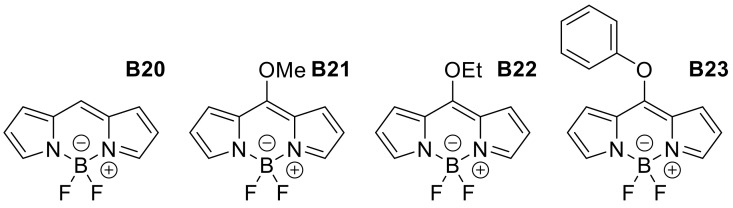
Alkoxy groups placed at the meso position.

**Figure A6:**
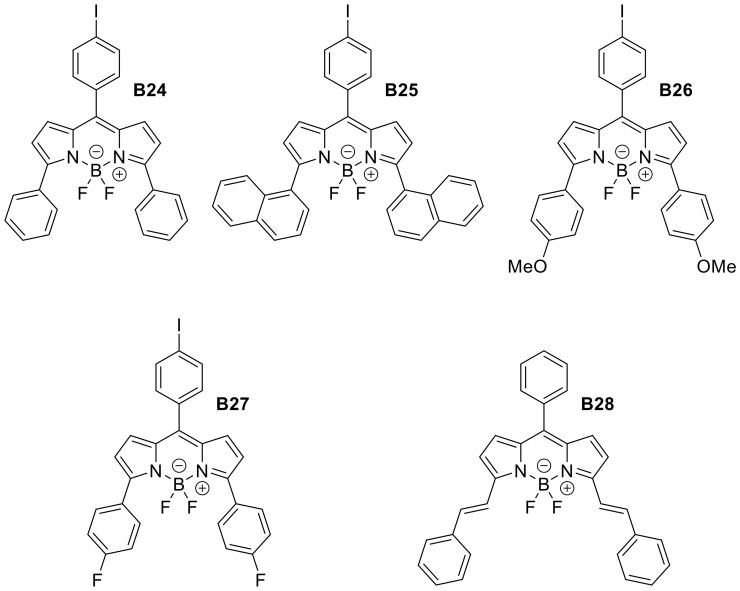
Aromatic groups placed at the meso and α positions.

**Figure A7:**
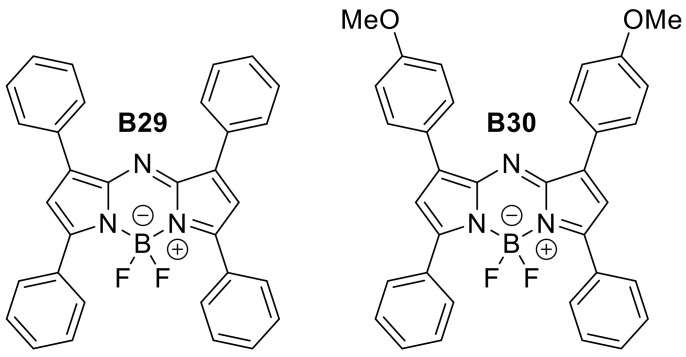
azaBODIPY with substituents at α and β positions.

**Figure A8:**
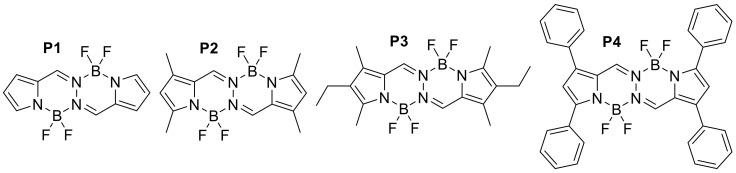
Various BOPHYs.

**Figure A9:**
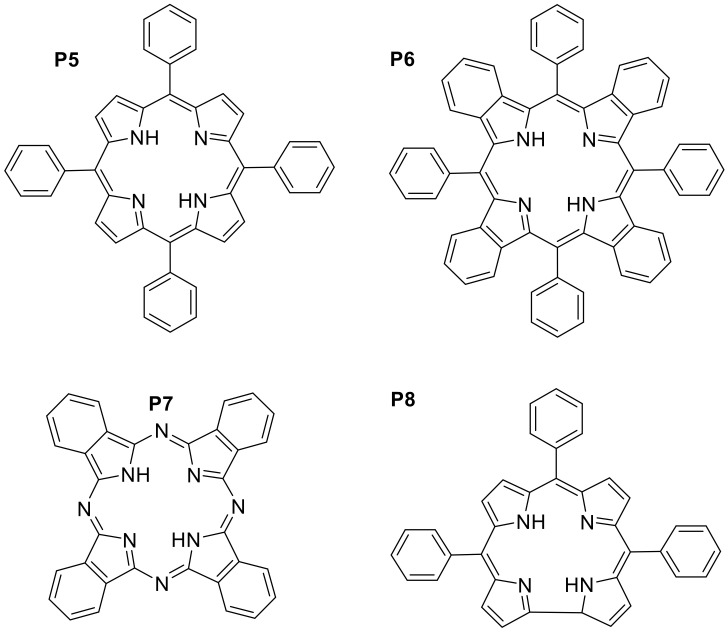
Porphyrinoides.

**Figure A10:**
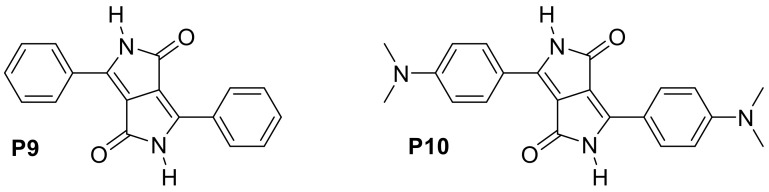
Diketo-pyrrolopyrroles.

All investigated structures and experimental parameters were selected from ref [50] and categorized into five families (Figure 1 and Table 1): hydrogen in meso and *α* and *β* substitution (**B1**–**5**), aromatic in meso and α and β substitution (**B6**–**9**), substitution on aromatic position (**B10**–**B13**), meso-amino derivatives (**B14**–**B19**), meso-*alkoxy* derivatives (**B20**–**23**) and others (**B24**–**B30**) containing BODIPYs with extended pi-systems composed by α-vinyl, β-conjugated and aza-BODIPYs. A test group (Table 2) is also described to verify the robustness of our model composed with 10 representative pyrrole-based dyes (**P1**–**P10**). The full representation of these structures is placed in Figure A1, Figure A2, Figure A3, Figure A4, Figure A5, Figure A6, Figure A7, Figure A8, Figure A9 and Figure A10.

## 3. Results and Discussion

### 3.1. Optimisation of the BODIPYs Structures and Orbitals Parameters

Optimization processes were carried out with different functionals, respectively the one used for the TD-DFT calculation. No imaginary frequencies were observed for any of the investigated molecules nor for any functionals used assessing the correct energy minimization of the structure. Regarding structural features, most of the BODIPY core are planar or quasi planar with small deviation up to 30 degree in extreme cases. This issue has already been addressed by Orte and coworkers [27] and, in our case, did not explicitly interfere with the TD-DFT calculations for the estimation of the 0-0 transition.

**Figure 2:**
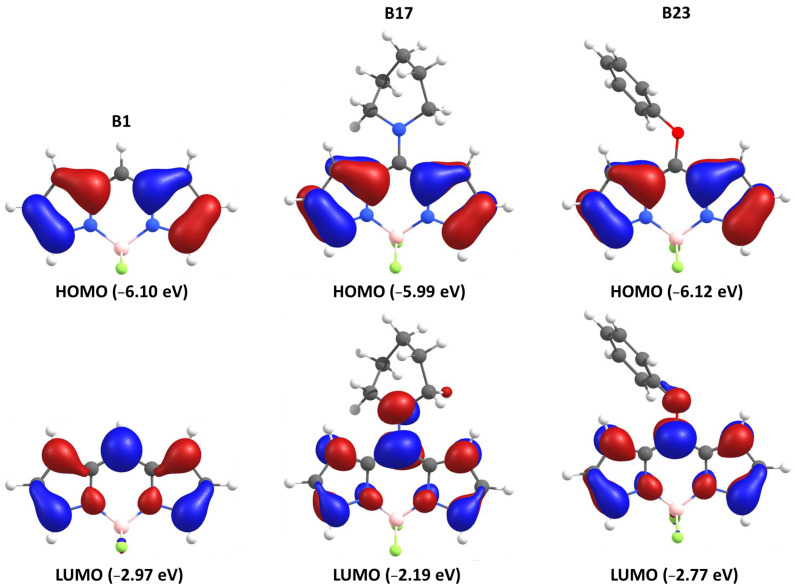
Comparison of the HOMO and LUMO contours for three selected investigated BODIPY’s.

The frontier molecular orbitals (HOMO (Highest Occupied Molecular Orbital) and LUMO (Lowest Unoccupied Molecular Orbital)) were generated and checked in order to verify that the correct modeling of the S_1_ state was achieved. Figure 2 depicts the HOMO-LUMO levels and MO contours for **B1**, **B17,** and **B23**. The HOMO-LUMO gaps are in good agreement with the BODIPY family standard values [50]. Generally, the HOMOs are built upon on the π-systems located on the pyrrole rings. Concurrently, the LUMOs are partially located on the meso-position accompanied by a depletion of the pyrrole π-systems relative to that found for the HOMOs. Minor contributions are also computed on the two fluorides of the BF_2_ group. When bulky groups, aromatics or conjugated systems are present at the meso-position, the LUMOs tend to extend towards these groups directly linked or through a heteroatom (i.e., oxygen or nitrogen). This interaction could be described as an intramolecular partial charge transfer.

### 3.2. TD-DFT Assessing the First Excited State

**Figure 3:**
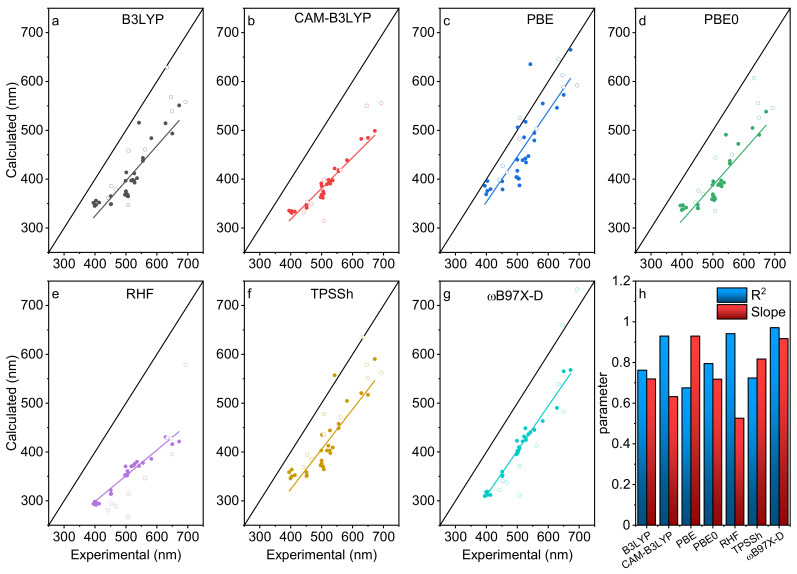
(**a**–**g**) graphs representing the calculated positions of the 0,0 S_0_ → S_1_ absorption peaks against the experimental values for various DFT computational methods. The straight black line represents the cases where the experimental values are equal to the theoretical ones. The dark dots (•) are the training series (**B1–B30**) and the empty circles (◦) are the test series (**P1–P10**). (**h**) linear regression parameters taken into consideration of the benchmarks. The experimental data are from Table 1 and Table 2, and the appropriate solvent field has been applied.

The lowest energy spin-allowed electronic transitions calculated from TD-DFT computations have been correlated with the maximum 0-0 peak in the absorption experimental spectra. Figure 3 shows these correlations compared with the ideal case where these values are identical. The computed values are systematically lower than the experimental, which is consistent with the literature [35,36].

To pin down what computational method appears the best, least square regressions have been performed on each dataset. Each of them consists of the lowest energy spin-allowed electronic transitions obtained from TD-DFT (as described in the computational details) and the positions of the maximum intensity peak (i.e., 0-0) in UV–vis spectra. Two parameters have been extracted: the R^2^ correlation and the slope. A R^2^ value approaching one implies a perfect or quasi-perfect correlation between computed and experimental wavelengths. Concurrently, a slope approaching one means that the deltas between each molecular species are the same numerical values. The “sensitivity” would be ideal in this case. Figure 3h displays these correlation parameters after a linear regression for each method for all 30 species. ωB97X-D seems to be the best suitable functional with the BODIPY dyes along with CAM-B3LYP as well as RHF methods.

**Figure 4:**
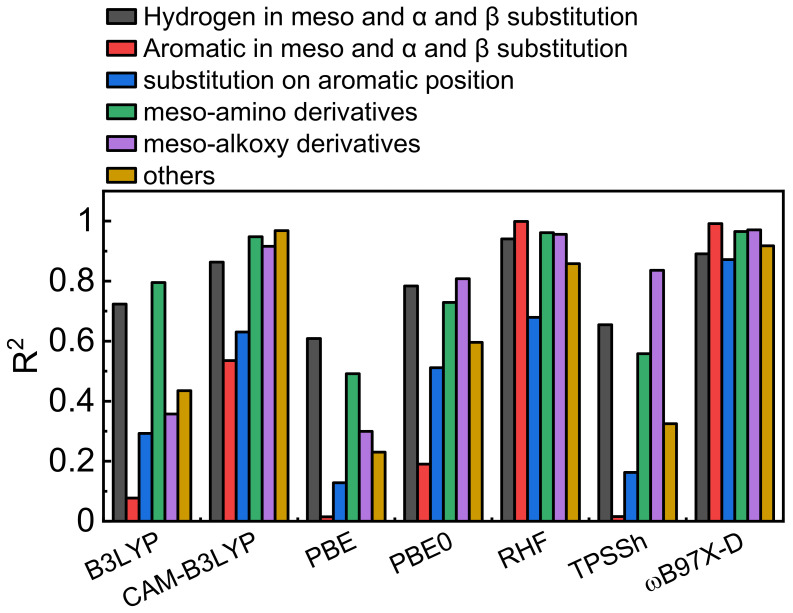
Correlation between R^2^ and the computational method for each family of BODIPY derivatives. Dark grey: No substituent placed at the meso, α, and β positions. Red: Aromatic groups placed at the meso, α, and β positions. Blue: Various substitutions placed on the aromatics. Green: Amino groups placed at the meso position. Brown: Other cases.

Figure 4 regroups the correlation parameters R^2^ separated by BODIPY groups as defined in “Investigated Structures” section. The CAM-B3LYP and ωB97X-D functionals as well as the RHF method appear to give results with good correlation with experiments (blue bars). The presence of an aromatic group (at the meso position in these series) has the greatest impact on the correlation mainly for the B3LYP, PBE, and PBE0. This, with the fact that ωB97X-D functional is giving the best results, indicates that the long-distance electronic correlation is important for this purpose. Indeed, this functional includes a 100% long-range exact exchange in its definition. For the investigated dyes, this interaction seems to be primordial during the interaction between light and a conjugated system and the formation of the charge transfer S_1_ state. In comparison with a previous study on generic organic molecules, a more exact long-range term is needed (~25% vs. 100%) [58]. In the cases of the B3LYP, PBE, PBE0, and TPSSh and RHF methods, low R^2^ values are obtained for molecules containing aromatic groups placed at the meso and *α* and *β* positions and substituents at various positions on the aromatic groups (**B10**–**B13**). However, an investigation as whether the inclusion or exclusion of specific groups on the BODIPY skeleton have any effect, has been examined. However, this endeavor was inconclusive. Adding or removing dyes has a neglectable effect on the resulting fits (R^2^ and slope) for each series. These tests have been performed by removing one dye at the time and examining what the effect on R^2^ and slope are (Table A2).

### 3.3. Model Validation

In order to validate this computational model obtained with the training series (dark dots; (**B1**–**B30**), a test group (**P1**–**P10**) represented in Figure 3 by empty circles were added to the graphs. The test group is composed of analogous ring-fused and larger cyclic pyrrole-based dyes such as BOPHYs (bis(difluoroboron)1,2-bis((1H-pyrrol-2-yl)methylene)hydrazine (**P1**–**P4**)), free base porphyrinoides (tetraphenyl porphyrin **P5**, tetrabenzonporphyrin **P6**, phtalocyanine **P7,** and corrole **P8**) and diketo-pyrrolopyrroles (**P9**–**P10**) with common substituents to ensure a high degree of diversity. With little to no surprise, the best fits were again observed for the CAM-B3LYP and the ωB97X-D functionals as well as the RHF method. For B3LYP, PBE, and PBE0, the results are spread too widely to consider them as adequate functionals for this purpose.

### 3.4. The “Fudge Factor” Approach

**Figure 5:**
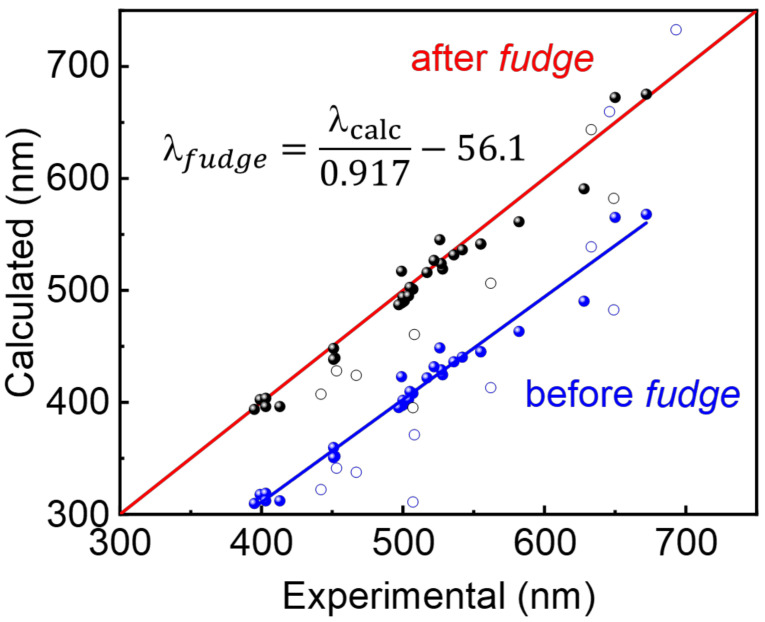
Linear correction obtained upon the use of the ωB97X-D with 6.311g (d,p) basis set.

Regarding the deviation of the slopes from the theoretical perfect theoretical match (black line in Figure 3), similarly to many other research groups, a “fudge factor” correction is also applied. This approach is generally accepted and is motivated on the ratio computation time vs quality of the method permitting to achieve a correlation slope close to 1 with a maximal R^2^. Based on the large computational investigation above, ωB97X-D with 6.311g (d,p) basis set appear the most appropriate for the largest set of different substrates (Figure 4) and were selected to test the *fudge factor* (Figure 5). The calculated data set was plotted on Figure 5 with a R^2^ of 0.97 and a slope of 0.917. After a mathematical correction of the data set, a simple linear equation was established in order to achieve a reasonable precision and accuracy. This simple correction procedure is a common method used throughout the literature and allows to estimate absorption 0-0 peak position with quick and low cost calculations [18,38,49]. The resulting sought equation is placed inside Figure 5.

## 4. Materials and Methods

The computations have been carried out with the Gaussian16 package [59] and ORCA 4.2.0 [60]. The calculations consisted in a simple three step-procedure. The geometries of all 30 BODIPYs and related pyrroliques dyes were preoptimized in their ground state with the B3LYP functional in conjunction with the 6-311g(d,p) basis set, as they are generally robust parameters for organic molecules in the literature. The optimized geometries were taken as starting points for optimization with solvent model (CPCM [61]) with each functional described below. Finally, TD-DFT computations were performed using the basis set 6-311g(d,p) in conjugation with def2-TZVP (Valence triple-zeta basis set) for heavy atoms. More precisely, B3LYP [62,63], CAM-B3LYP [64], PBE [65], PBE0 [66], TPSSh [67], and ωB97X-D [68] in addition of the RHF method [69] were used. Geometries were kept fixed during the TD-DFT computations and the excited state of interest (first excitation state, N = 1) was retrieved for each molecule. DFT integration grid was set to 4 and the final grid was set to 5. All other parameters were kept at their default values. Table A1 regroups all numerical data calculated for each method. The fudge correction was performed in two steps: first the calculated parameters were plotted against the experimental ones and fitted with a linear regression. Slope, R^2^ and intercept were obtained. Second, the equation of the linear regression was then equalized with the x = y diagonal to obtain a translation equation.

## 5. Conclusions

An improved computational methodology for the prediction of the low-energy absorption peak of the BODIPY dyes has been developed. This method appears as a promising method with a lot of potential for applications because it is simple, cost effective and relatively accurate. As stated in the Introduction, many studies have addressed this important problem, but a good all-around method was not yet available. A wide benchmark series of TD-DFT methods, namely, to examine a large spread of possible wavelength values in the absorption spectra of a series of 30 BODIPYs, was employed and then validated with a test group of 10 related structures. The investigated BODIPYs included anchored groups such aromatics, electro-acceptor/donor substituents and saturated carbons chains to ensure that the design methodology could be applied in a more general manner. The best results were obtained using the ωB97X-D functional (with the basis set 6-311g(d,p)) giving the best correlation parameters (R^2^ > 0.97). Concurrently, this study also pointed out that the electronic correlation at long distance is important to describe the BODIPYs first excited state. However, in the classic situation for a need of anticipated absorption maximum of the S_0_ → S_1_ transition for the BODIPY dyes, the “fudge factor” approach is a relatively affordable (i.e., best ratio accuracy vs computational resources) method (meaning relatively acceptable predicted values for relatively short computational time).

## Figures and Tables

**Table 1:** Investigated BODIPYs structures and experimental parameters (λ_max_ abs, λ_max_ em and Φ) from ref [50].

Compound	R_1_	R_2_	R_3_	R_4_	Solvent	λ_max_ abs (nm)	λ_max_ em (nm)	Φ
**Hydrogen in Meso and α and β Substitution**								
**B1**	H	H	H	H	EtOH	499	535	0.93
**B2**	H	H	H	Me	EtOH	507	520	0.81
**B3**	H	Me	H	Me	EtOH	505	516	0.80
**B4**	H	Me	Me	Me	EtOH	528	535	0.56
**B5**	H	Me	Et	Me	EtOH	517	546	0.70
**Aromatic in Meso and α and β Substitution**								
**B6**	Phenyl	H	H	H	CH_2_Cl_2_	500	527	0.03
**B7**	2,4,6-trimethylbenzene	H	H	H	CH_2_Cl_2_	501	521	0.84
**B8**	2,4,6-trimethylbenzene	Me	H	Me	AcOEt	500	508	0.92
**B9**	2,4,6-trimethylbenzene	Me	Et	Me	CH_2_Cl_2_	526	535	0.72
**Substitution on Aromatic Position**								
**B10**	2,4,6-trimethoxybenzene	Me	Et	Me	AcOEt	527	535	0.86
**B11**	2,6-didecyloxybenzene	Me	Et	Me	EtOH	522	536	0.82
**B12**	2,6-dichlorobenzene	Me	Et	Me	CH_2_Cl_2_	536	548	0.65
**B13**	1,3-di-tert-butylbenzene	Me	H	Me	CH_2_Cl_2_	499	507	0.97
**Meso-Amino Derivatives**								
**B14**	H	H	H	H	MeOH	497	507	0.87
**B15**	NH_2_	H	H	H	MeOH	399	437	0.92
**B16**	NMe_2_	H	H	H	MeOH	395	438	0.09
**B17**	Piperidine	H	H	H	MeOH	413	537	0.001
**B18**	*N*-aniline	H	H	H	MeOH	403	461	0.16
**B19**	*N*-phenylmethanamine	H	H	H	MeOH	403	453	0.09
**Meso-alkoxy derivatives**								
**B20**	H	H	H	H	Cy	504	511	0.96
**B21**	OMe	H	H	H	Cy	452	487	0.84
**B22**	OEt	H	H	H	Cy	451	487	0.96
**B23**	OPh	H	H	H	Cy	451	486	0.88
**Others**								
**B24**	4-iodobenzene	H	H	Ph	CHCl_3_	555	588	0.20
**B25**	4-iodobenzene	H	H	1-napthalene	CHCl_3_	542	607	0.38
**B26**	4-iodobenzene	H	H	PhOMe	CHCl_3_	582	626	0.42
**B27**	4-iodobenzene	H	H	4-fluorobenzene	CHCl_3_	555	590	0.22
**B28**	Phenyl	H	H	CH=CH_2_Ph	CH_3_CN	628	642	0.84
**B29**	-	Ph	H	Ph	CHCl_3_	650	672	0.34
**B30**	-	PhOMe	H	Ph	CHCl_3_	672	695	0.23

Me = Methyl, Et = Ethyl, AcOEt = Ethyl acetate, Ph = Phenyl, Cy = Cyclohexhane.

**Table 2:** Test group composed by related pyrrole-based dyes.

Compound	Solvent	λ_max_ abs (nm)	λ_max_ em (nm)	Φ	Ref
**P1**	CH_2_Cl_2_	442	465	0.95	[51]
**P2**	CH_2_Cl_2_	467	485	0.92	[51]
**P3**	CH_2_Cl_2_	453	497	1.00	[52]
**P4**	CHCl_3_	508	524	0.96	[52]
**P5**	Toluene	419	649	0.11	[53,54]
**P6**	Toluene	633	783	0.28	[55]
**P7**	Benzene	693	698	0.43	[56]
**P8**	CH_2_Cl_2_	416	671	0.14	[53,54]
**P9**	DMSO	507	519	0.74	[57]
**P10**	DMSO	562	580	0.57	[57]

## Data Availability

The data presented in this study are available upon request.

## Sample Availability

Not available.

## Appendix B

**Table A1:** Comparison of the experimental position of the 0-0 peak of the low-energy absorption band with the calculated ones for all investigated structures.

Compound	λ_max_ abs (nm)	B3LYP	CAM- B3LYP	PBE	PBE0	RHF	TPSSh	ωB97X-D	ωB97X-D fudge
**B1**	499	368	363.1	404.2	358.9	352.5	372.9	396.9	488.81
**B2**	507	364.9	370.4	387.3	359.5	357.5	364.6	408.12	501.05
**B3**	505	368.3	375	400.9	363.1	360.9	369.7	409.75	502.82
**B4**	528	393	390.8	434.2	385.3	375.7	397.7	424.65	519.07
**B5**	517	397.1	389.8	438.8	388	370.6	403	421.87	516.04
**B6**	500	375.3	368.2	417.3	366.9	351.9	382.5	397.65	489.63
**B7**	501	413.9	366.8	506	395.5	353.4	435.3	398.58	490.65
**B8**	500	374	385.9	439.9	369	351.8	377	401.89	494.25
**B9**	526	397	393.1	442.4	388.6	373.2	403	448.72	545.31
**B10**	527	411.8	394.9	517.4	397.4	373.9	444	429.12	523.94
**B11**	522	398.6	398.6	485.7	391.2	371.6	412.8	431.8	526.86
**B12**	536	401.9	397.5	447.3	393	379.9	409.2	436.21	531.67
**B13**	499	396.6	391.3	440.6	388.2	370.4	403	422.89	517.15
**B14**	497	368.1	363.2	404.2	359	352.6	372.8	395.37	487.15
**B15**	399	345.1	333.3	369	336.7	298.4	345.9	317.78	402.56
**B16**	395	351.6	335.5	386.2	345.8	293.9	358.8	309.67	393.72
**B17**	413	352	333.6	379.7	341.7	294.3	352.5	312.05	396.31
**B18**	403	355.9	334.6	396	346.7	296.7	363.8	318.81	403.68
**B19**	403	347.2	330.7	375.9	338.5	291.5	350.2	312.05	396.31
**B20**	504	365.8	362	400.9	357.1	351.7	370.3	402.67	495.11
**B21**	452	348.9	341.6	378.8	340.6	314.5	351.7	351.78	439.63
**B22**	451	348.7	341.5	395.7	340.5	313.9	350.5	350.67	438.42
**B23**	451	365	347.3	395.7	347.7	321.8	358.7	359.66	448.22
**B24**	555	437.6	416.5	479.2	431.7	378.2	448.6	445.17	541.44
**B25**	542	515.5	421.9	635.3	491.1	371.3	557.3	440.39	536.23
**B26**	582	483.9	438.8	554.9	472.4	385.9	504.6	463.41	561.32
**B27**	555	443.5	418.5	494.5	437.5	377.6	457.7	445.2	541.47
**B28**	628	514.3	482.3	546.1	504.7	430.4	520.5	490.36	590.70
**B29**	650	493.5	484.7	572.7	490.7	416	517	565.21	672.30
**B30**	664	551	498.9	664.8	538.3	421.3	590.5	567.93	675.27
**P1**	442	361.6	332	398.7	352	280.4	369.2	322.12	407.29
**P2**	467	379	348.8	413.8	370.1	289.9	385.9	337.65	424.22
**P3**	453	385.7	353.8	427.6	376.4	294.2	394.3	341.29	428.19
**P4**	508	458.2	398.8	525.6	444	314.5	477.7	371.07	460.66
**P5**	646	567.9	550.6	613	556	427.8	578.7	659.64	775.25
**P6**	633	628.8	-	645.4	606.9	428.9	635.7	538.87	643.59
**P7**	693	558.3	555.6	592.2	545.6	578.4	562.6	732.8	855.01
**P8**	649	538.9	-	588.6	525.9	395.5	551.2	482.58	582.22
**P9**	507	346.8	314.1	482.4	335.4	267.5	436.5	311.06	395.23
**P10**	562	460.7	419	503.2	450.4	346.1	470.8	413.11	506.49

## Appendix C

**Table A2:** Variation of the parameters R^2^ and slope when adding or removing one dye at the time from the training data set for each computational method.

Removed Dye	B3LYP		CAM- B3LYP		PBE		PBE0		RHF		TPSSh		ωB97X-D	
	Slope	R^2^	Slope	R^2^	Slope	R^2^	Slope	R^2^	Slope	R^2^	Slope	R^2^	Slope	R^2^
None	0.72	0.76	0.63	0.93	0.93	0.68	0.72	0.79	0.53	0.94	0.82	0.72	0.92	0.97
**B1**	0.72	0.77	0.63	0.93	0.93	0.68	0.72	0.80	0.53	0.94	0.81	0.73	0.92	0.97
**B2**	0.72	0.77	0.63	0.93	0.93	0.69	0.72	0.80	0.53	0.94	0.82	0.74	0.92	0.97
**B3**	0.72	0.77	0.63	0.93	0.93	0.69	0.72	0.80	0.53	0.94	0.82	0.73	0.92	0.97
**B4**	0.72	0.77	0.63	0.93	0.94	0.68	0.72	0.80	0.52	0.94	0.82	0.73	0.92	0.97
**B5**	0.72	0.76	0.63	0.93	0.93	0.68	0.72	0.80	0.52	0.94	0.82	0.73	0.92	0.97
**B6**	0.72	0.77	0.63	0.93	0.93	0.68	0.72	0.80	0.53	0.94	0.82	0.73	0.92	0.97
**B7**	0.72	0.76	0.63	0.93	0.93	0.69	0.72	0.79	0.53	0.94	0.82	0.73	0.92	0.97
**B8**	0.72	0.77	0.63	0.93	0.93	0.68	0.72	0.80	0.53	0.94	0.82	0.73	0.92	0.97
**B9**	0.72	0.77	0.63	0.93	0.93	0.68	0.72	0.80	0.52	0.94	0.82	0.73	0.91	0.98
**B10**	0.72	0.76	0.63	0.93	0.92	0.68	0.72	0.79	0.52	0.94	0.81	0.72	0.92	0.97
**B11**	0.72	0.76	0.63	0.93	0.93	0.68	0.72	0.80	0.52	0.94	0.82	0.72	0.92	0.97
**B12**	0.72	0.77	0.63	0.93	0.94	0.68	0.72	0.80	0.52	0.94	0.82	0.73	0.92	0.97
**B13**	0.72	0.76	0.63	0.93	0.93	0.67	0.72	0.79	0.53	0.95	0.82	0.72	0.92	0.98
**B14**	0.72	0.77	0.63	0.93	0.93	0.68	0.72	0.80	0.53	0.94	0.81	0.73	0.92	0.97
**B15**	0.74	0.76	0.65	0.93	0.94	0.66	0.74	0.79	0.53	0.94	0.84	0.72	0.92	0.97
**B16**	0.75	0.77	0.65	0.94	0.96	0.68	0.75	0.80	0.52	0.94	0.85	0.73	0.92	0.97
**B17**	0.73	0.76	0.64	0.93	0.94	0.67	0.73	0.79	0.52	0.94	0.83	0.72	0.91	0.97
**B18**	0.74	0.77	0.64	0.93	0.96	0.68	0.74	0.80	0.52	0.94	0.85	0.73	0.92	0.97
**B19**	0.74	0.76	0.64	0.93	0.95	0.67	0.74	0.79	0.52	0.94	0.84	0.72	0.92	0.97
**B20**	0.72	0.77	0.63	0.94	0.93	0.68	0.72	0.80	0.53	0.94	0.82	0.73	0.92	0.97
**B21**	0.71	0.76	0.63	0.93	0.92	0.67	0.71	0.79	0.52	0.94	0.81	0.72	0.91	0.97
**B22**	0.71	0.76	0.63	0.93	0.93	0.67	0.71	0.79	0.52	0.94	0.81	0.72	0.91	0.97
**B23**	0.72	0.76	0.63	0.93	0.93	0.67	0.72	0.79	0.52	0.94	0.81	0.72	0.92	0.97
**B24**	0.72	0.76	0.63	0.93	0.94	0.68	0.72	0.79	0.53	0.94	0.82	0.72	0.92	0.97
**B25**	0.70	0.83	0.63	0.93	0.89	0.77	0.70	0.84	0.53	0.94	0.79	0.81	0.92	0.97
**B26**	0.70	0.75	0.63	0.93	0.91	0.66	0.70	0.79	0.53	0.94	0.80	0.71	0.93	0.97
**B27**	0.72	0.76	0.63	0.93	0.93	0.67	0.71	0.79	0.53	0.94	0.81	0.72	0.92	0.97
**B28**	0.69	0.73	0.61	0.93	0.95	0.66	0.69	0.77	0.51	0.94	0.81	0.70	0.95	0.98
**B29**	0.73	0.74	0.62	0.92	0.95	0.65	0.72	0.77	0.54	0.94	0.83	0.70	0.89	0.97
**B30**	0.67	0.70	0.62	0.91	0.84	0.59	0.68	0.74	0.56	0.95	0.75	0.65	0.91	0.96
